# Vaginoscopic Incision of Oblique Vaginal Septum in Adolescents with OHVIRA Syndrome

**DOI:** 10.1038/s41598-019-56471-2

**Published:** 2019-12-27

**Authors:** Chunxia Cheng, Jigyasa Subedi, Aiqian Zhang, Grace Johnson, Xingping Zhao, Dabao Xu, Xiaoming Guan

**Affiliations:** 1 Department of Obstetrics and Gynecology, Third Xiangya Hospital, Central South University, Changsha, Hunan China; 2 Department of Obstetrics and Gynecology, Baylor College of Medicine, Houston, TX USA

**Keywords:** Reproductive disorders, Urogenital reproductive disorders

## Abstract

This study is to evaluate the efficacy and safety of vaginoscopic incision of oblique vaginal septum in adolescents with Obstructed hemi-vagina and ipsilateral renal agenesis (OHVIRA) syndrome. It is about Fourteen adolescents with OHVIRA syndrome managed by vaginoscopic incision of the oblique vaginal septum using a “No-touch” technique over an 8-year period. In all fourteen adolescents with OHVIRA the oblique vaginal septum was incised successfully without any intraoperative complications. Postoperative pain was unremarkable and each patient’s symptoms resolved postoperatively. The 3-month postoperative follow up office vaginoscopy revealed that the vaginal septum had not reformed nor was any vaginal stenosis noted. Vaginoscopic incision of the oblique vaginal septum using a “No-Touch” technique is a safe, minimally invasive, and effective approach for treating OHVIRA syndrome in adolescents with hematocolpos. This technique may be utilized to minimize disruption to the undeveloped vaginal wall and postoperative pain while providing excellent surgical visualization throughout the procedure.

## Introduction

Obstructed hemi-vagina and ipsilateral renal agenesis (OHVIRA) syndrome is a rare Mullerian duct anomaly that presents with an obstructing vaginal septum, ipsilateral renal anomalies and various types of uterine malformations^1,2^. The prevalence of congenital Mullerian anomalies is reported in up to 25% of women with a history of primary infertility^3^, in up to 6.7% of the general population, and in up to 0.4% to 1% in an unselected population^4,5^. OHVIRA syndrome encompasses a variety of clinical presentations including an obstructing hemi-vagina combined with various Mullerian and renal anomalies such as a uterus didelphys, unilateral obstructed hemi-vagina, ipsilateral renal agenesis, dysplastic kidney, pelvic kidney, or ectopic ureter^1,6^. In addition, there are rare variants of OHVIRA syndrome, such as with complete septate uterus^7^. The triad of obstructed hemi-vagina, uterus didelphys and ipsilateral renal anomaly was named Herlyn-Werner-Wunderlich syndrome (HWWS) in 1980. This classification was further delineated by Rock and Jones into three separate categories comprising HWWS^8,9^. Type I: Blind hemi-vaginal septum without an opening. In this case the uterine horn behind the septum has no connection to the outside nor into the contralateral uterus and menstrual blood accumulates in the cavity behind the vaginal septum. Type II: Blind hemi-vaginal septum with an opening. In this case there is a pinpoint-size hole in the septum through which a limited amount of menstrual blood drains out. The ipsilateral uterus is separate from the other horn. Type III: Complete hemi-vaginal septum with cervical fistula. In this case a fistula connects the two cervices of the obstructed vagina and the contralateral cervix.

Patients usually present after menarche with progressive dysmenorrhea, lower abdominal pain, a paravaginal mass, foul mucopurulent discharge, and intermenstrual bleeding due to hemi hematocolpos^10,11^. Accurate identification is important as a delay or lack of treatment may increase the risk of endometriosis, pelvic adhesions and infertility^2,11,12^. The diagnosis of OHVIRA syndrome requires a multi-modal approach, integrating the patient’s history, clinical presentation, imaging studies including ultrasonography (USG), computed tomography (CT), magnetic resonance imaging (MRI), and direct visualization via hysteroscopy or laparoscopy^13–15^.

Regarding options for surgical treatment, a single stage procedure in which the septum is either resected or completely divided has been reported in the literature^16^. For the adult patient with OHVIRA syndrome, vaginoplasty is typically performed under direct visualization with the help of a speculum or vaginal retractors. However, in adolescent patients these operations require wide exposure of the immature vagina using vaginal retractors which often results in disruption or even tearing of the vaginal walls causing postoperative pain. Vaginoscopic management using a “No-touch” technique might be an optimal option for adolescent patients. Little has been reported regarding a surgical approach for adolescents with OHVIRA syndrome whose vagina is immaturely developed^1^. Previously, we have developed a hysteroscopic management of OHVIRA with hematocolpos using a “No-touch” technique in virgin adolescents. This procedure has several benefits including excellent safety outcomes, improved visualization, decreased post-operative pain due to no vaginal retractors or speculum use, and the ability to leave the hymen intact^17^. In this paper, we review a series of 14 cases of patients treated with the above procedure to validate its efficacy and safety.

## Methods

We retrospectively analyzed fourteen adolescents with OHVIRA syndrome who underwent hysteroscopic incision of an oblique vaginal septum according to the technique described above from 2009 to 2017. These adolescents range in age from 10 to 19 years old and none were yet sexually active. Their characteristics are summarized in Table 1. The most common presenting complaint was dysmenorrhea. All cases had double cervices. Twelve patients had didelphic uteri, and two patients had a complete septate uterus. All patients had a solitary kidney. Seven patients had right kidney intact with left renal agenesis and seven patients had left kidney intact with right renal agenesis. According to Rock and Jones’ classification criteria^8,9^, seven patients were diagnosed with type-I, six with type-II, and one with type-III malformation (Table 1). The preoperative diagnosis of OHVIRA syndrome was made by clinical presentation, transabdominal and transrectal ultrasound scan, CT or MRI, and routine digital rectal examination. Pain was measured with self-rating assessment and an observational scoring scale between 0 and 10.

**Table 1:** All Fourteen Patients’ profile of the OHVIRA syndrome.

case	Age/Menarche	Preoperative pain scale	History of		Postoperative Pain scale	Uterus Didelphys	Double Cervix	Complete Septate Uterus	Type
			DYS	CF					
1	19/13	Severe	Y	N	None	Y	Y	N	Type-II
2	13/13	Mild	N	N	None	Y	Y	N	Type-I
3	15/14	Mild	N	N	None	Y	Y	N	Type-II
4	13/12	Mild	N	N	None	N	Y	Y	Type-III
5	11/11	Mild	N	N	None	Y	Y	N	Type-I
6	11/11	Moderate	Y	N	None	N	Y	Y	Type-I
7	10/10	Mild	Y	N	None	Y	Y	N	Type-I
8	13/12	Moderate	Y	N	None	Y	Y	N	Type-II
9	12/11	Severe	Y	N	None	Y	Y	N	Type-I
10	12/11	Severe	Y	N	None	Y	Y	N	Type-II
11	19/13	Severe	Y	N	None	Y	Y	N	Type-I
12	19/11	Severe	Y	Y	None	Y	Y	N	Type-II
13	14/12	Severe	Y	N	None	Y	Y	N	Type-I
14	19/14	Mild	Y	N	None	Y	Y	N	Type-II

OHVIRA: Obstructed hemivagina and ipsilateral renal agenesis, Pain score; 0-none, 1–2 mild, 3–6 moderate, 7–10 severe, DYS: Dysmenorrhea, CF: Concurrent fever, N: No, Y: Yes.

**Figure 1:**
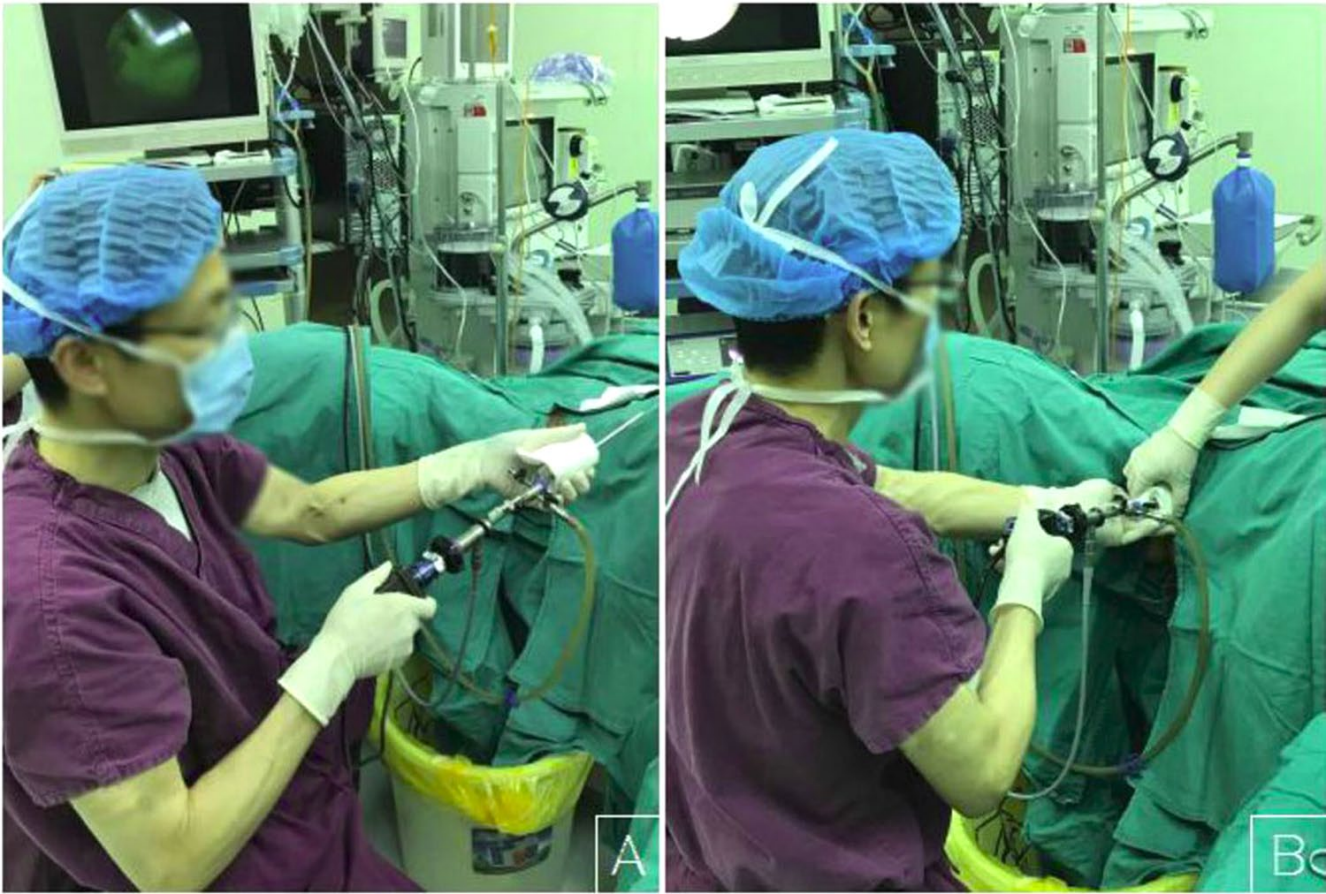
(**A**) Put a cotton gauze pad (red arrow) around the hysteroscope; (**B**) An assistant held a cotton gauze pad (white arrow) around the hysteroscope and applied pressure toward the orifice of the vagina during the vaginoscopy.

**Figure 2:**
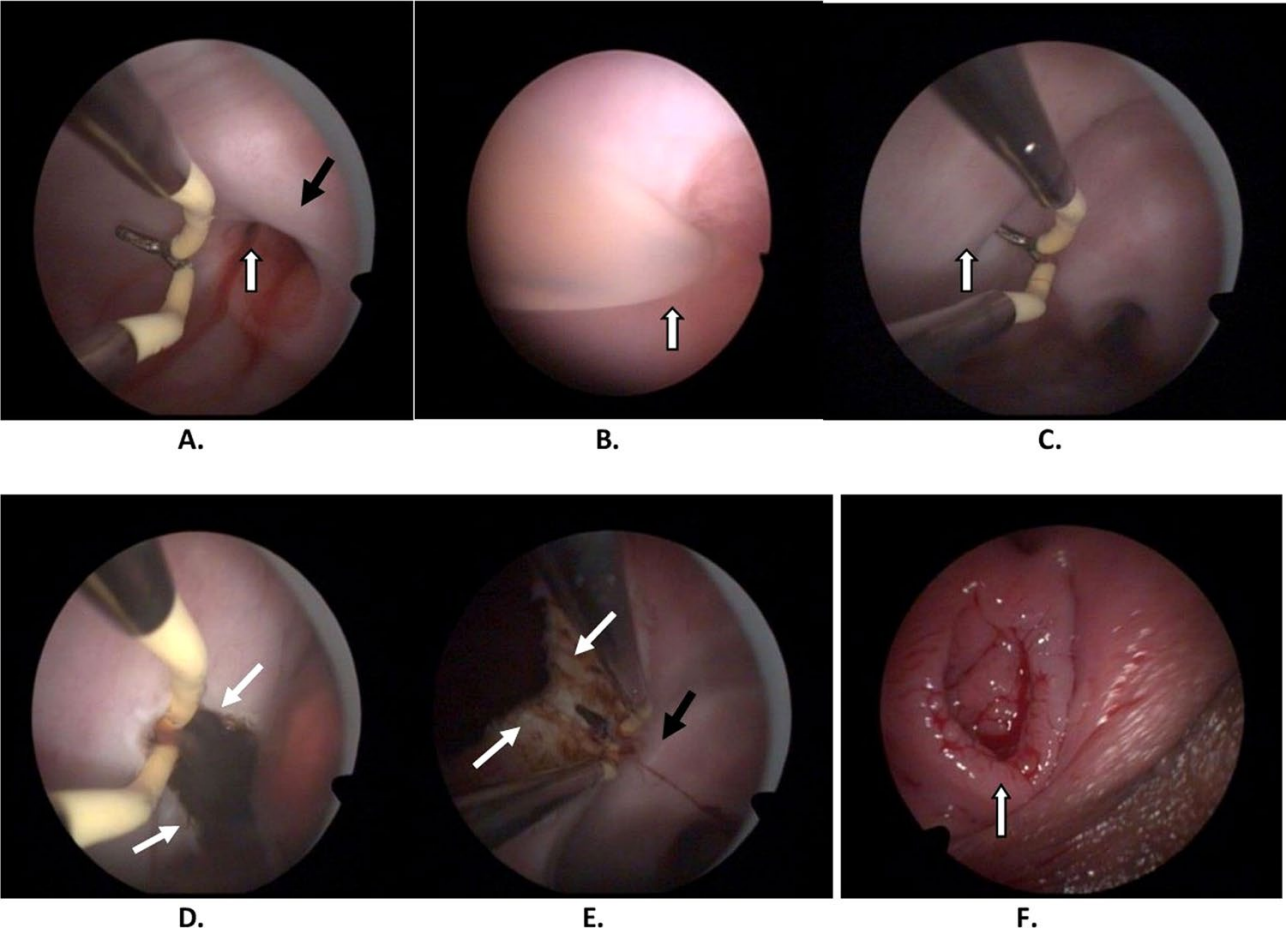
(**A**) Vaginoscopic view of cervical canal with spilling of blood (white arrows), left cervix (black arrow). (**B**) Vaginoscopic view of cervical fistula catheterization (white arrow). (**C**) Bulging of septum after 5% mannitol injection (white arrow). (**D**) Hysteroscopic incision at most prominent portion of bulge (white arrows). (**E**) Upper part of septum has been incised; left cervix visualized (right cervix not visualized due to distance of hysteroscope). (**F**) An intact hymen after the surgery.

**Figure 3:**
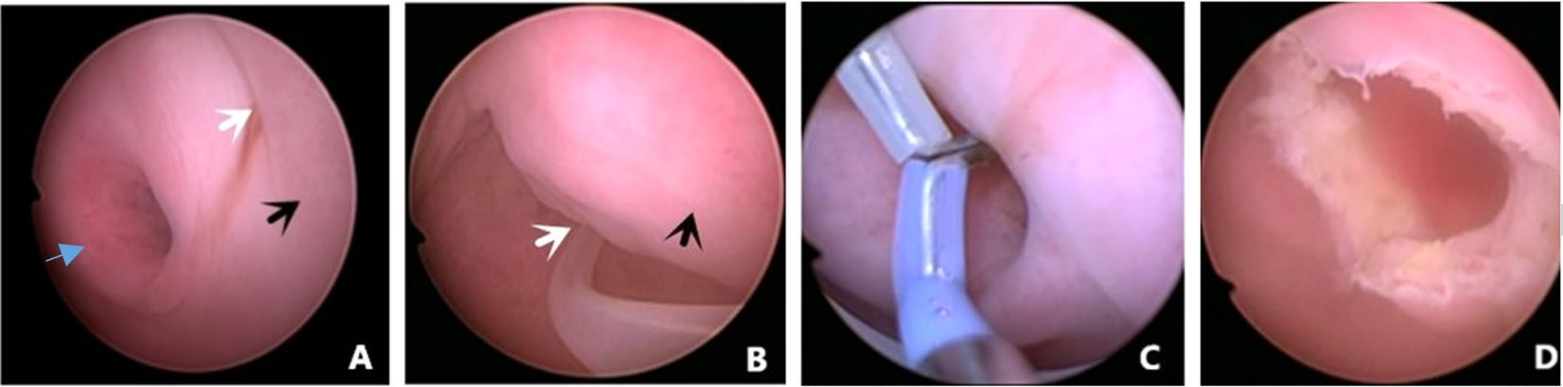
Hysteroscopic incision of a high location of type II left oblique vaginal septum. (**A**) A hole (white arrow) in the septum, the septum (black arrow) was not obvious at vaginoscopy using a hysteroscope because of the high location of the septum and the excellent drainage of menstrual blood from behind the septum; right cervix (blue arrow); (**B**) Hysteroscopic catheterization used to inject normal saline into the cavity behind the septum to further identify the border of the septum (black arrow) through the hole (white arrow); (**C**) A L-hook bipolar electrode (Olympus company, Japan) ready to incise the septum longitudinally beginning at the hole in the septum; (**D**) The left septum had been incised completely, revealing a very high, small septum.

**Figure 4:**
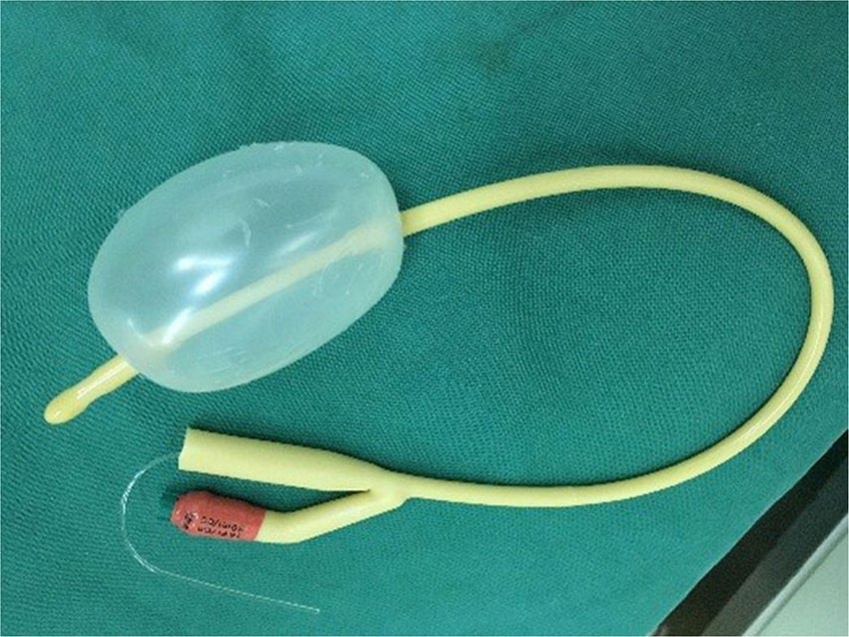
A 14 Fr Foley catheter (used in this study) with 80 ml air inside the balloon.

The procedure is outlined as follows: after induction of anesthesia, the bladder is distended with normal saline to improve visualization by transabdominal ultrasound during the procedure. A diagnostic 4.5mm-outer sheath hysteroscope (Olympus Company, Japan) is inserted into the vagina cautiously through the hymenal ring^18^. An assistant holds a cotton gauze pad around the hysteroscope and applies pressure toward the orifice of the vagina to decrease the outflow of the distension media while the operator manipulates the hysteroscope (Fig. 1). Simultaneously, the distention pressure is set to 150 mmHg and the inflow rate of the media is set to 450 mL/min in order to achieve adequate vaginal distention during the vaginoscopy. In eleven of the fourteen patients, the vaginal septum was easily identified by either a bulge protruding from the lateral vaginal wall or a pinpoint-size hole in the vaginal septum. In the remaining three patients, the lack of an obvious bulge made it difficult to distinguish the vaginal septum from the vaginal wall (Figs. 2A and 3A). As these patients had previously had menses, it is hypothesized that the menstrual blood must have drained via a cervical fistula between the two cervical canals (Fig. 2A) or via the small hole in the septum (Fig. 3A). On this basis, a hysteroscopic catheter was threaded through the cervical canal and into the cervical fistula under direct visualization of a hysteroscope and transabdominal ultrasound (Fig. 2B). Approximately 50–100 mL of either 5% mannitol or normal saline (depending on whether unipolar or bipolar resectoscope was used) was then instilled into the blind vaginal pouch via the cervical fistula. This caused the vaginal septum to bulge into the vagina to further differentiation it from the surrounding vaginal wall (Fig. 2C). This ability to backfill the occluded pouch is also very helpful when operating on patients with a particularly high septum that is difficult to locate (Fig. 3). Under ultrasound guidance, an 8 mm~9 mm outer sheath unipolar or bipolar resecto-hysteroscope fitted with an L-hook electrode was introduced into the vagina and a longitudinal incision was made at the most prominent bulge of the vaginal septum (equipment from different manufacturers were used during these cases which included: Olympus Company, Japan; Wisap Company, Germany; or Karl-Storz Company, Germany). The incision may then be extended cephalad to approximately 3~5 mm below the cervices (Fig. 2D,E) and caudad to the junction of the oblique septum and the lateral vaginal wall resulting in incision of the entire length of the septum. Thick chocolate-like fluid from retained menstrual blood was then allowed to drain from behind the vaginal septum. The cervix previously hidden by the vaginal septum may then be visualized. A 16 Fr Foley catheter (Guangzhou Weili Medical Instruments Limited Company, Guangdong, China) was then inserted between the incised halves of the oblique septum and the balloon was inflated with up to 80 ml air under direct view of hysteroscopy (Fig. 4). The exact level of air instilled is determined by the size needed to achieve adequate separation of the incised tissue. In cases where the septum is very high or the cavity behind the septum very small, less volume may be required (Fig. 3). The Foley catheter is left in place for two days to prevent postoperative adhesions and reformation of the vaginal septum. Patients are only limited in regards to very active movements for the two days following their surgery in order to prevent displacement of the vaginal balloon. Two days following the surgery, the Foley balloon is deflated and removed. All patients are followed up for one year postoperatively either in the outpatient department or by phone.

### Ethics approval and informed consent

Approval given to the study by The Institutional Review Board (IRB) of Third Xiangya Hospital and Xiangya Hosptial, Central South University. The procedure was performed in accordance with relevant guidelines and regulations. Informed consent was obtained after the procedure was fully explained from all participants and their legal guardians.

## Results

All patients underwent a trans-abdominal ultrasound guided vaginoscopic incision of an oblique septum using a “No-touch” technique^19^. The procedures were unremarkable and did not need to transfer to a traditional “open” surgery. The operation time for each case totaled 20 to 40 minutes with minimal blood loss. There were no surgical complications. After the procedure, 10 patients who had previously reported dysmenorrhea reported complete relief of dysmenorrhea. Notably, all patients that underwent this procedure reported no post-operative pain. The hymen remained intact for all patients (Fig. 2F).

**Figure 5:**
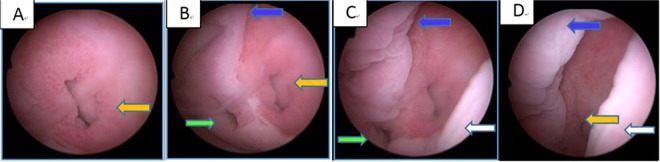
A left OHVIRA patient’s 3 months postoperative following-up vaginoscopy using a diagnostic hysteroscope, the distance from cervix to hysteroscope was getting farther from (**A–D**). left cervix (yellow arrow), right cervix (green arrow), trace of the septum on the anterior vaginal wall (blue arrow) and posterior vaginal wall (white arrow).

All patients were successfully followed up for one year postoperatively either in the outpatient department or by phone. The initial 11 patients (out of 14 discussed here) underwent vaginoscopy using a diagnostic hysteroscope 3 months postoperatively^19^, which revealed neither stenosis nor adhesions between the incisions of the oblique vaginal septum. The vaginal wound healed very well and no additional procedures were deemed necessary for these individuals (Fig. 5). Throughout the one year follow up period, none of the patients experienced recurrence of their symptoms. Meanwhile, a follow up ultrasound confirmed that the vaginal septum had not reformed in any of the patients.

## Discussion

OHVIRA syndrome is typically diagnosed at puberty, presenting with cyclic, lower abdominal pain resulting from long-standing, retained menstrual blood in the obstructed hemi-vagina^1^. Although the exact etiology of this syndrome is not known, it is hypothesized that it represents an aberrancy of Mullerian as well as Wolfian duct development^19^. The actual incidence of Mullerian duct anomalies is not known ranging from 0.1% to 3.5% depending on the study^20,21^. Of these Mullerian anomalies, the incidence of OHVIRA syndrome ranges from 0.16 to10%^22,23^.

Once the diagnosis of OHVIRA syndrome is made, surgical management should be carried out as soon as possible in order to relieve symptoms and to prevent long term complications related to retrograde menstrual flow including hematocolpos, pyocolpos, endometriosis, and pelvic adhesions. In situations in which surgical correction is not readily available or contraindicated for other reasons, menstrual suppression with hormonal medications is a reasonable alternative, however, only surgery provides definitive management. In our series, as the reader will note, 4 patients with OHVIRA type II were 19 years old at the time of their surgery-remote from menarche. Ideally these patients would have had their procedures closer to menarche, but due to the health care disparities in rural communities, they did not present to our facility until their late teen years. This is not uncommon as this syndrome is exceedingly rare and patients with Type II and III present with fewer symptoms than Type I patients in which the oblique septum is completely obstructive.

Previously reported surgical approaches for correction of OHRIVA syndrome involved excision of the vaginal septum with drainage of the obstructed fluid^1^, excision of the vaginal septum with closure of the vaginal defect by suturing together the mucosal surfaces^24^, use of a spinal needle for aspiration of the hematocolpos followed by extension of the incision to identify the margins of the septum and hemi-vagina^25^, and use of a vaginal mold or stent postoperatively in cases with a high, thick septum^26^. The conventional surgical approaches involve retractors, scissors, scalpels, and sutures. These techniques require wide exposure of the vagina, which often results in disruption of the hymen and the vagina or even tearing of the vaginal walls associated with increased postoperative pain. Despite the use of retractors, visualization often remains a challenge during these surgeries.

In a word, the technique we reported here are as follows: (1) Diagnostic vaginoscopy and hysteroscopy of uterine cavity to further confirm the diagnosis, (2) Vaginoscopic incision of the oblique vaginal septum using a hysteroscopic L-hook electrode, and finally, (3) Placement of a 14 Fr or 16 Fr Foley catheter into the previously obstructed hemi-vagina with inflation of the Foley balloon with 50~80 ml (varying with the size of the oblique septum) air to prevent adhesions of the incised oblique vaginal septum. To the best of our knowledge, it is the first case series that focuses on the safety and efficacy of this technique. We believe our vaginoscopic approach using the “No-touch” technique provides a superior, minimally invasive technique well suited for adolescents with often times narrow, underdeveloped vaginas with intact hymens. It allows excellent surgical visualization compared to traditional techniques that rely on vaginal retractors, allowing for relatively simple incision of even the highest septum (Fig. 3). One of the patients in our series had a very high left oblique septum with a small hole through which menstrual efflux drained. A hysteroscopic catheter was threaded through this hole and distension media was instilled into the cavity causing the septum to balloon into the vagina, allowing for simple hysteroscopic judgement and incision under excellent visualization (Fig. 3). Additionally, as no vaginal retractors are used, post-operative pain is virtually non-existent.

Regarding safety and efficay, our procedure avoids many of the common complications of this type of surgery. Common complications include postoperative adhesions, tearing of the vaginal wall, reformation of the obstruction and vaginal stenosis. In our procedure the septum is incised by use of a hysteroscope, rather than excised as occurs during traditional surgery. Incision is preferred over excision as the latter may lead to scar formation and is more likely to be complicated by re-obstruction and vaginal stenosis^27^. The Foley catheter balloon placement between the incised vaginal septum is a novel technique that prevents future adhesions. More rare complications were also entirely avoided in our case series including organ injury (to the ureter, rectum, hymen, bladder, or cervix), excessive bleeding or fluid overload.

Though not necessarily relevant to all populations, the ability to maintain hymenal integrity is an important benefit of our procedure for certain populations, such as in China, where there is high importance placed on the integrity of the hymen by both patients and their families, especially during the teenage period. The cultural norms surrounding hymenal integrity are very different than those in the west with complexity created by personal and cultural values related to sexuality, religion, and self-determination^28–31^.

In summary, our case series study suggests that vaginoscopic incision of the oblique vaginal septum is a safe, minimally invasive, and effective approach for treating OHVIRA syndrome in adolescent patients with hematocolpos. This technique is well suited for adolescent patients with undeveloped vaginal walls because it minimizes postoperative pain while it optimizes surgical visualization.

## Data Availability

No datasets were generated or analyzed during the current study.
